# Characterization of *vanA*-harboring plasmids supports differentiation of outbreak-related and sporadic vancomycin-resistant *Enterococcus faecium* isolates in a tertiary care hospital

**DOI:** 10.1186/s12866-025-04058-5

**Published:** 2025-05-28

**Authors:** A. Sobkowiak, N. Scherff, V. van Almsick, F. Schuler, T. J. Brix, A. Mellmann, V. Schwierzeck

**Affiliations:** 1 Institute of Hygiene, University Hospital Münster, Münster, Germany; 2 Department of Cardiology I – Coronary and Peripheral Vascular Disease, Heart Failure, University Hospital Münster, Münster, Germany; 3 Institute of Medical Microbiology, University Hospital Münster, Münster, Germany; 4 Institute of Medical Informatics, University of Münster, Münster, Germany

**Keywords:** Vancomycin-resistant *Enterococcus faecium* (VREfm), Molecular surveillance, Long-read whole genome sequencing (lrWGS), Plasmid, *vanA*, Transmission, Hospital

## Abstract

**Background:**

The prevention of vancomycin-resistant *Enterococcus faecium* (VREfm) infections and transmissions poses a major challenge to hospitals. Vancomycin resistance can be plasmid encoded; however, as the analysis of plasmids is challenging, so far only a few reports have provided a detailed characterization of plasmids in nosocomial VREfm transmission. Here we describe a nosocomial VREfm outbreak caused by a *vanA* positive ST80 isolate. *vanA* plasmid sequence data was used to distinguish outbreak-associated isolates from sporadic VREfm cases and to investigate the spread of this plasmid within the local VREfm population.

**Methods:**

446 VREfm isolates were collected from routine surveillance between 01/2022 and 02/2024 and analyzed using long-read whole genome sequencing (lrWGS). Genetic relatedness of isolates was evaluated based on core genome multilocus sequence typing (cgMLST). Genetically similar *vanA* plasmids were identified using a Mash based approach.

**Results:**

30 genetically similar VREfm isolates were identified in patients’ screening and environmental samples. Infection control evaluation confirmed transmission through shared hospital rooms. All outbreak-related VREfm isolates, including environmental samples, carried a highly similar *vanA* plasmid (Mash distance of < 0.001) with an identical replicon type. After enhanced infection control measures were established, no new transmissions were detected. Comparison with additional VREfm isolates from the respective department showed no evidence for further plasmid transmission.

**Conclusions:**

Our study illustrates how *vanA* plasmid analysis can support the evaluation of VREfm transmission in hospitals. The outbreak-associated *vanA* plasmids were genetically highly similar, but could be clearly distinguished from other *vanA* plasmids in the local hospital population. Taken together, detailed analysis of hospital-associated *vanA* plasmids can improve our understanding of VREfm transmission and epidemiology.

**Supplementary Information:**

The online version contains supplementary material available at 10.1186/s12866-025-04058-5.

## Background

In July 2024, the Centers for Disease Control and Prevention (CDC) published new surveillance data concerning the antimicrobial resistance threats in the United States from 2021 to 2023 [1]. Here a significant increase in hospital onset vancomycin-resistant *Enterococcus faecium* (VREfm) infections was described. The most recent European antimicrobial resistance surveillance network (EARS-Net) report also describes a worrying increase in VREfm isolates in Europe [2]. VREfm poses a major concern to healthcare systems worldwide, as the bacteria can cause serious infections and frequently initiate nosocomial outbreaks [3], despite well-established surveillance efforts [4]. In recent years, whole genome sequencing (WGS) has become the gold standard to investigate potential outbreaks in the hospital setting [5]. Unfortunately, the discriminatory power of current genomic analysis methods for VREfm appears to be lower than for other multi-drug resistant organisms [6], probably due to its monomorphic population structure.

Although vancomycin resistance can be plasmid encoded, little is known about the role of these plasmids in nosocomial transmissions [6, 7], because it has previously been difficult to analyze plasmids [8, 9]. The recently developed long-read whole genome sequencing (lrWGS) technology enables the assembly of long DNA fragments including plasmids as part of routine molecular surveillance. This analysis can add an additional dimension of genetic information to outbreak investigations [10].

For this reason, we investigated a nosocomial VREfm outbreak event in our tertiary care hospital in Münster, Germany, using lrWGS technology. We characterized the outbreak-associated vancomycin resistance plasmid and compared this *vanA* plasmid to the local *vanA* plasmid landscape of our hospital. Our results demonstrate that lrWGS can be used to characterize *vanA*-harboring plasmids as part of a routine molecular outbreak investigation and facilitates – based on plasmid sequence comparisons—the differentiation of outbreak-related isolates from sporadic cases.

## Material and methods

### Setting and study design

VREfm isolates were collected from screening swabs and clinical specimens at the University Hospital Münster (UHM) between 01/2022 and 02/2024. The UHM is a 1,450-bed tertiary-care hospital in Münster, Germany. In line with national recommendations, all high-risk patients (i. e., hemato-oncological patients) and patients with known VREfm colonization or infection are screened for VREfm [11]. In addition, Enterococci cultured from relevant clinical specimen such as biopsies, urine or primarily sterile body fluids undergo antimicrobial susceptibility testing. As part of a routine molecular surveillance strategy, isolates of patients that have tested positive for VREfm for the first time were sequenced by lrWGS. Only one isolate per patient was sequenced.

### Microbiological methods

Rectal swabs were cultivated on VRE selective agar plates (VRESelect Agar, BioRad, Hercules, USA) and incubated at 5% CO_2_ and 36 C° ± 1 C° for at least 28 h. Clinical specimen were cultured on Columbia agar with 5% sheep blood (Becton Dickinson, Heidelberg, Germany) and incubated at 36 C° ± 1 C° up to 72 h. Bacterial species were identified using matrix-assisted laser desorption/ionization time-of-flight mass spectrometry (MALDI-TOF/MS) (MALDI Microflex® LT or MALDI Biotyper Sirius one IVD System, Bruker, Bremen, Germany) with identification scores above 2.0. Antimicrobial susceptibility testing was performed using the EUCAST disk diffusion test or a Vitek2 automated system (bioMérieux®, Marcy l′Étoile, France) applying the 2022 EUCAST clinical breakpoints. Environmental sampling was performed using polywipes (mwe, Corsham, Wiltshire, UK). Swabs were incubated in Tryptic Soy Broth containing lecithin tween (Merck Millipore, Eppelheim, Germany) for 24 h at 37 °C. Samples were plated on Columbia blood agar (Thermo Scientific, Schwerte, Germany) and VRE selective agar (VRESelect, Bio-Rad) and incubated for 28 h at 36 C° ± 1 °C. Vancomycin resistance was confirmed for all isolates by presence of the *van* genotype (eazyplex®VRE, amplex, Gars-Bahnhof, Germany).

### Whole genome sequencing methodology

Genomic DNA of bacterial isolates were extracted using either Monarch® Genomic DNA Purification Kit (New England Biolabs, Ipswich, MA, USA) or ZymoBIOMICS 96 MagBead DNA Kit with Lysis tubes (Zymo Research, Freiburg, Germany) as described in the manufacturer's protocol and sequenced on a PacBio® Sequel IIe system (Pacific Biosciences, Menlo Park, CA, USA) using the SMRTbell® Express Template Prep Kit 2.0 (Pacific Biosciences Inc., Menlo Park, CA, USA). Sequencing Data was assembled *de novo* using the SMRT® Link software suite 11 with default parameters. For core genome multilocus sequence typing (cgMLST), the published cgMLST scheme for *E. faecium* was used [12]. The following quality control and exclusion criteria were applied: duplicates, other species and contaminated samples were excluded as well as sample with low data quality (presence of cgMLST targets < 95%), an average coverage < 15 and samples without *van* resistance genes.

### Analyses of WGS data

After lrWGS and *de novo* assembly, the genomes were further analyzed by different tools implemented in Ridom SeqSphere^+^ software version 10 (Ridom GmbH, Münster, Germany). We characterized the genetic relationship of the isolates using multilocus sequence typing (MLST), sequence type (ST) and, clonal complex (CC) assignment for a broader grouping of genotypes. The cgMLST [12] and the complex type (CT) were assigned in accordance to the cgMLST database (www.cgMLST.org) for high discrimination of genotypes. MLST STs were identified using the scheme for *E. faecium* [13] implemented in Ridom SeqSphere^+^ (Ridom GmbH). Antimicrobial resistance (AMR) genes and their location were determined using target gene sets based on NCBI AMRFinderPlus [14]. Based on the allelic profiles from cgMLST [12]; we constructed a minimum-spanning tree to characterize the genetic relationships between isolates. Based on previously published results pairs of isolates with a cgMLST distance of ≤ 3 were followed up by the infection control team to identify potential transmission cases [15].

To analyze plasmids, contigs were further characterized to predict replicon type, relaxase type and mobility using MOB-suite software 3.1.8 [16] and all plasmids were compared and clustered using Mash (21 k-mers, sketch size 10,000, Mash distance ≤ 0.001) [17, 18]. Annotations were performed using Bakta 1.10.3 and the database v.5.1.0 [19].

Forty-two additional VREfm isolates were used to construct a database for a comprehensive comparison of *vanA* plasmids associated with the outbreak event as well as local plasmids.

### Visualization

Minimum-spanning trees were created with Ridom SeqSphere^+^ (Ridom GmbH) based on cgMLST [12] allelic distances. The plasmid map of the annotated representative outbreak associated plasmid was created with SnapGeneViewer® 7.2 (SnapGene® software, Dotmatics; available at snapgene.com). The plasmid comparison was aligned with blast v2.16.0 [20] and visualized by pyGenomeViz v1.5.0 with *pgv-gui* [21] using 1,000 bp as the minimum comparison length.

### Statistics

All statistical analysis was performed using basic R v. 4.4.0 [22] and package *dplyr*.

### Epidemiological evaluation of the outbreak

Patient data were evaluated by infection control staff based on patients′ medical records and interviews with nursing or medical staff on site. In addition, the following criteria were used:

1. Contact within the department: the patient pair was admitted to the department, overlapping for at least one calendar day within the hospital admission.
2. Ward contact: the patient pair was admitted to the same ward, overlapping for at least one calendar day within the hospital admission.
3. Room contact: the patient pair was admitted to the same room, overlapping for at least one calendar day within the hospital admission.

## Results

### Characteristics of VRE isolates during study period

As a first step we wanted to capture the current epidemiology of VREfm at our hospital. During an observation period of 26 months, 446 VREfm isolates collected at the UHM were sequenced by lrWGS. Of these, 255 VREfm had a *vanA* genotype (57.17%) and 189 a *vanB* genotype (42.38%); two isolates harbored a combined *vanA/B* genotype. Isolates were detected in screening samples (*n* = 375, 84.08%) as well as in different clinical samples (15.92%). The median age of patients was 65 years (IQR 55–73) and most patients were male (*n* = 273, 61.21% vs. *n* = 173, 38.79%). MLST analysis revealed that 55% of the isolates belong to ST80 (*n* = 245) and 40% to ST117, respectively (Supp. Table 1). The majority of ST80 had a *vanA* genotype (85.10%) and ST117 were predominantly a *vanB* genotype (82.54%) (Supp. Table 1). The *vanA/B* isolates belong to ST117/CT71 and ST80/CT6045.

### Detailed analysis of VREfm outbreak event

**Fig. 1:**
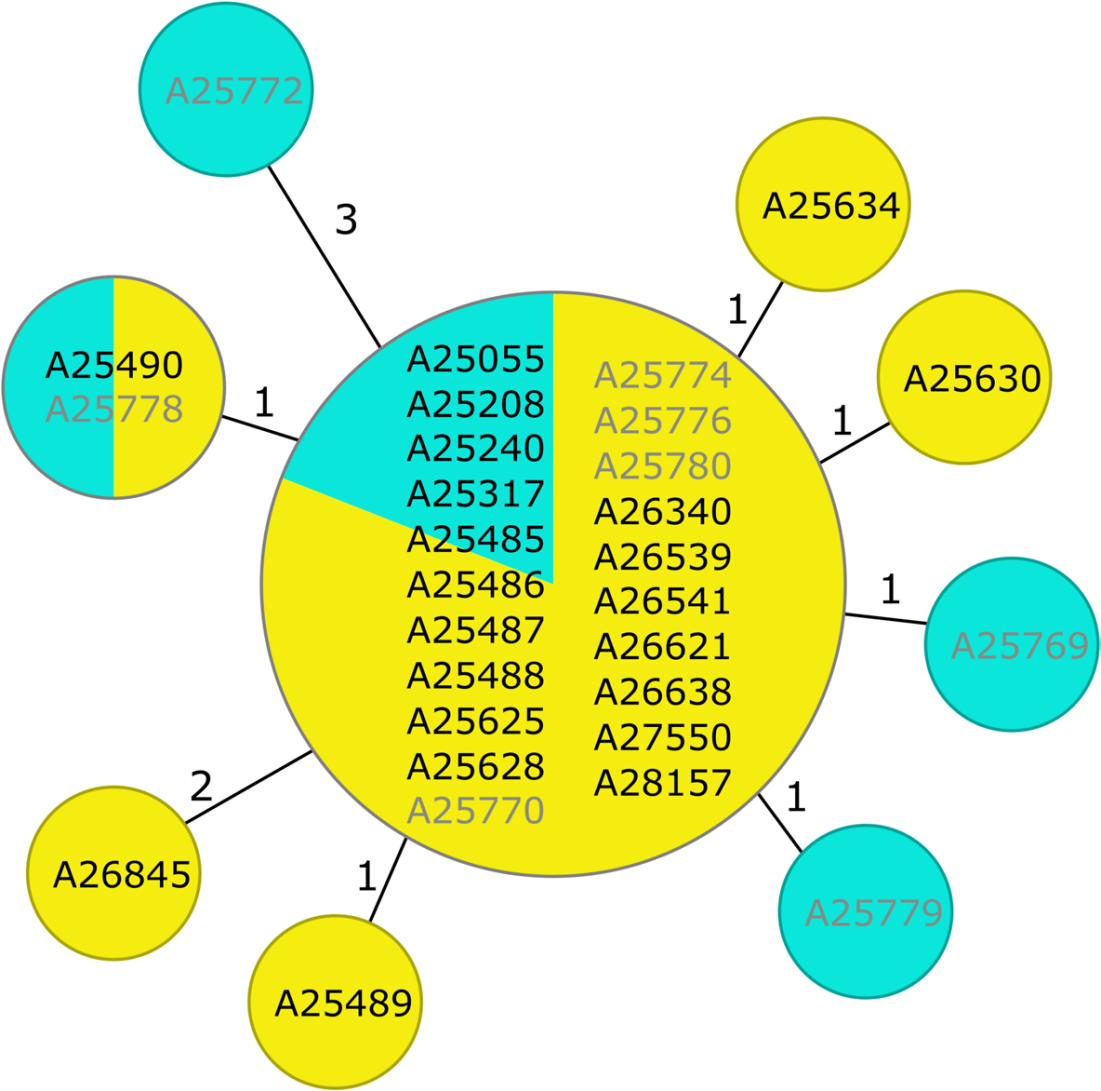
Minimum Spanning Tree of the outbreak-associated VREfm isolates and environmental samples The tree is constructed based on cgMLST allelic profiles, where missing cgMLST targets were pairwise ignored and showed the genetic relationship of the outbreak-associated isolates. Isolates within a circle have identical cgMLST allelic profiles, colors within the circles show the proportion of isolates’ source (yellow: patient sample; cyan: environmental sample). Numbers on connecting lines between two circles, i. e. genotypes, indicate allelic differences. Each VRE isolate is represented by a sample ID and coloring indicates the source patients’ isolates are in black letters (n = 22) and environmental samples in grey letters (n = 8)

**Fig. 2:**
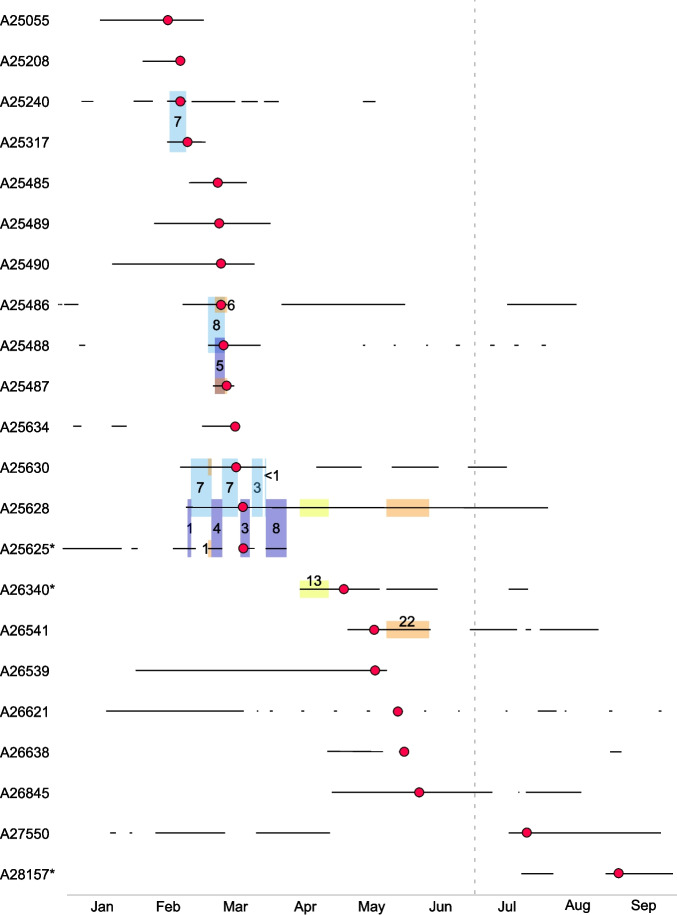
Timeline of the VREfm outbreak event Black lines illustrate the hospital stay of each patient during the observation period in 2022. Each sample ID is connected to one isolate and one patient. Red dots indicate the time the outbreak-associated isolates were detected. Colored boxes of the same color connect patients, which shared a room during hospital admission. Numbers in or next to the boxes indicate the time occupying a shared room (in days). An asterisk next to the sample ID indicates non-nosocomial isolates

In the beginning of 2022, we noticed an increase of *vanA* positive VREfm isolates from neighboring wards. In total, 22 VREfm isolates were identified in screening samples between February 2022 and September 2022. Out of 22 colonized patients, nine were female and 13 male with a median age of 64 (IQR 53 -67). Nineteen cases were identified as nosocomial. As the situation fulfilled epidemiological outbreak criteria, we initiated an outbreak investigation [23]. All isolates were subjected to lrWGS to investigate if isolates were genetically closely related and a clonal transmission within the hospital admission seemed possible. The isolates were characterized as ST80/CT1470 and CC17, harboring a *vanA* positive plasmid. As part of infection control measures, we performed environmental sampling from the affected ward in March 2022. We sampled high-touch surfaces or areas exclusively frequented by healthcare workers (Supp. Table 2). Here, we could detect eight VREfm from environmental sampling that shared the same ST and *vanA* genotype indicating possible indirect transmissions through surfaces. cgMLST of all 30 isolates confirmed close genetic relationship (Fig. 1). Detailed epidemiological evaluation corroborated with the genotypic information and revealed connections for possible nosocomial transmission routes in most cases, as many patients shared a room during their hospital stay (Fig. 2).

Next, we analyzed the plasmids of these 30 isolates. In total, the isolates contained 165 plasmids. Regarding to *vanA* genes, four isolates contained two *vanA* positive plasmids, leading to a total number of 34 plasmids that harbor *vanA*. The majority of *vanA* gene clusters comprised all associated genes, i. e. *vanA*, *vanH*_*A*_, *vanR*_*A*_, *vanS*_*A*_, *vanX*_*A*_, *vanY*_*A*_, *vanZ*_*A*_ (Supp. Figure 1). Most *vanA* plasmids had a size of 23.9 kb, were predicted non-mobilizable and contained no other antibiotic resistance genes. The replicon type was identified as rep_cluster_889. In addition, the *vanA* plasmids of the outbreak-associated isolates showed Mash distances ≤ 0.001, indicating a high degree of similarity.

### Comparison with other *vanA* positive isolates

**Fig. 3:**
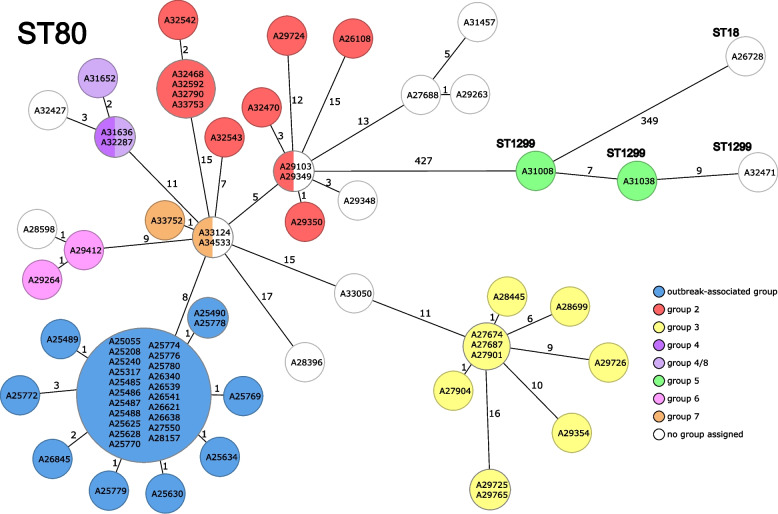
Overview of the identified *vanA* plasmid groups during the observation period cgMLST based minimum spanning tree of VREfm isolates from the department during a 26 months observation period (n = 42) and the outbreak-associated isolates (n = 30) are illustrated. Each VREfm isolate is represented by a sample ID. The numbers next to the connecting lines indicate the cgMLST allelic differences between the isolates. Eight vanA plasmid groups (see also Table 1) were identified and are indicated by color. The outbreak-associated genotypes are colored in blue and show, for example, for A25055 a Mash distance of 0.0103 compared to cgMLST-based neighboring isolates (i. e., A33124). Of note, isolate A31636 contains two vanA plasmids assigned to plasmid group 4

After enhanced infection control measures, including intensified surface disinfection, were established on the affected ward, no further transmission of this particular VREfm clone was detected. Next, we wanted to investigate if there was any ongoing transmission of the *vanA* carrying plasmid associated with this outbreak event. Hence, we compared the outbreak-associated isolates with 42 additional VREfm isolates from the same department collected during the entire observation period. These isolates match the overall population of *vanA* VREfm isolates at our hospital (Supp. Table 1). Overall, the comparative isolates harbored 58 *vanA* plasmids and could be categorized into eight plasmid groups including the outbreak-associated plasmids (Fig. 3). Of these, 14 comparative isolates harbored two or three *vanA* plasmids.

cgMLST analysis revealed a minimal allelic distance of 8 alleles to the outbreak-associated isolates. Interestingly, plasmids associated with the outbreak event could be clearly distinguished from comparative ones (Table 1, Supp. Figure 2, Supp. Table 3). The maximum Mash distance within the outbreak-associated VREfm cluster was 0.001, while the most similar plasmids of the comparison isolates showed Mash distances between 0.0077 to 0.0103 (Supp. Table 4) compared to the outbreak-associated plasmid group. For example, a Mash distance of 0.0077 differentiated the plasmids of the outbreak-associated plasmid group to the plasmid of isolate A32543 from plasmid group 2. Therefore, we did not find any evidence of ongoing plasmid outbreak after the clonal transmission event.

**Table 1:** Plasmid groups (containing > 2 isolates) and plasmid characteristics

plasmid group#	number of plasmids	size^+^	Antimicrobial resistance encoding genes	replicon type	relaxase type	predicted mobility
1	31	23.9 kb	vanA*, vanH_*A*_*, vanR*_A_*, vanS_*A*_*, vanX*_*A*_*, vanY*_*A*_*, vanZ*_*A*_	rep_cluster_889	none	non-mobilizable
2	11	30.3 – 30.8 kb	*vanA, vanH*_*A*_*, vanR*_*A*_*, vanS*_A_*, vanX_*A*_*, vanY*_*A*_*, vanZ*_A_*, aph(3')-IIIa, erm(B), ant(6)-Ia, sat4	rep_cluster_889	none	non-mobilizable
3	10	33.7 – 34.4 kb	*vanA, vanH*_A_*, vanR_A_*, vanS_*A*_*, vanX*_*A*_*, vanY*_*A*_*, vanZ*_A_*, aph(3')-IIIa*, erm(B), ant(6)-Ia*, sat4	rep_cluster_1763, rep_cluster_889	none	non-mobilizable
4	4	24.7 kb	*vanR*_A_*, vanS_*A*_, aph(3')-IIIa, erm(B), ant(6)-Ia*, sat4	rep_cluster_889	none	non-mobilizable

^+^size of complete circular plasmids only

*gene is missing in some plasmids

Until the time of writing, the plasmid detected in the outbreak isolates has not been identified in any database. The most similar plasmid in the database PLSDB was NZ_AP026775.1 from Hanoi in Vietnam with a Mash distance of 0.0061 (sketch size for DB search is 1,000) which is 28.5 kb larger (Supp. Figure 3). Even a blast search at NCBI only detected plasmids that are over 4.0 kb larger than our outbreak-associated plasmid with only 23.9 kb (Supp. Figure 3). The first three hits at NCBI were CP059749.1, CP059767.1, MW821651.1.

## Discussion

Here we present a retrospective analysis of the VREfm population in a German tertiary care hospital between 2022 and 2024 and report a detailed cluster investigation concerning a VREfm clone including the analysis of its *vanA* plasmid.

In our dataset of 446 VREfm isolates, the majority were *vanA* positive (57.17%). This result is in line with other national reports and neighboring European countries, where a switch to *vanA* positive VREfm has been observed after *vanB* positive isolates had dominated the population for many years [24–26]. Overall, the epidemiological data described in our study matches observations from other hospitals in Germany and Europe. Therefore, our data appears to be a good representation of the hospital-associated VREfm population. Our report is based on a single hospital, yet we believe the conclusions are applicable to other hospitals with a similar VREfm epidemiology.

The reported outbreak event affected 22 immunocompromised patients, a well-known risk group for VREfm colonization and infection [27, 28]. The VREfm isolates associated with the event were identified as ST80/CT1470 *vanA* positive, a clone frequently involved in hospital transmission and outbreaks [29]. As ST80 is a common nosocomial VREfm strain in German hospitals, MLST typing is not sufficient to investigate nosocomial transmission chains [24, 30]. For this reason, we used the highly discriminatory cgMLST-based approach based on the allelic profiles of up to 1,423 target genes to identify potential transmission events. Previously published studies used cgMLST distances of ≤ 3 for VRE transmission clusters [15]. We used this cgMLST threshold as a first guide to identify potential transmission cases and followed these cases up with detailed contact tracing.

As we analyzed the lrWGS data of the isolates, we noticed the isolates were not only closely genetically related based on cgMLST, but also the “primary” *vanA* plasmid showed little sequence variation across all outbreak-associated isolates even in environmental samples (Fig. 3). The environmental samples were collected from surfaces or areas only frequented by health care workers to show that the detection of these VREfm is evidence for insufficient hand hygiene or surface cleaning. Genetically closely related VREfm isolates in this context are consistent with a clonal outbreak of the isolates through direct contact, i. e., shared rooms as well as through indirect contact, i. e., contaminated surfaces.

Because of the high genetic similarity and low mutation rate [31], the discriminatory power of established genomic analysis methods for VREfm is considered low and outbreak events can potentially be overestimated [15]. This poses a challenge for molecular based infection control investigations. Our results suggest that an analysis of *vanA* plasmids can support outbreak analysis, as identical plasmids present additional evidence for a close genetic relationship between isolates and clonal transmission. On the other hand, analysis of *vanA* plasmids can be used to rule out isolates. For example, there are several occasions where isolates are closely related based on cgMLST with an allelic distance of 0 or 1, but their plasmids differ markedly (A28598 [no plasmid group assigned], A34533 [no plasmid group assigned], A29349 [plasmid group 2]). This observation corroborated with our findings that there were neither direct contact between patients nor evidence of indirect transmissions via surfaces in these cases.

Occasionally, we identified plasmid groups, i. e., 2 and 3 that were present in isolates with higher cgMLST allelic distances (> 5 alleles). These cases might represent genetically stable *vanA* plasmids that are well-adapted in the VREfm population. It remains unknown, whether *vanA* plasmids are exchanged within the host between different strains of bacteria. Unfortunately, these questions cannot be answered with our dataset. Here, studies investigating a higher number of isolates per patient might be helpful to determine the impact of plasmids for the dissemination of VREfm.

After the enhanced infection control measures had been established, no genetically related VREfm isolates were detected in the meantime. Using a Mash based approach [18]; we found no evidence for horizontal gene transfer (HGT) or persistence of the plasmid in the affected department. This observation is in line with our characterization that the plasmid did not encode a relaxase and was therefore classified as “non-mobilizable” by MOB-suite (Table 1). HGT is regarded a key mechanism how antimicrobial resistance genes are transferred in Gram-negative enterobacteria [32, 33]. Currently less studies have investigated the HGT of *vanA* plasmids. For VREfm, smaller mobile genetic elements might also play an important role for the transfer of resistance genes. In fact, several studies only show the analysis of transposons containing *vanA* genes [34, 35] or evidence of HGT of Tn*1546*-like structures instead of plasmids [36, 37]. On this note, one limitation of our study is that it solely relies on in silico analysis, therefore, the mobility classification is based on the current state of knowledge, but has not been experimentally proven as part of this study. As Ares-Arroyo et al. have shown previously the number of as “non-mobilizable” characterized plasmids might be overestimated [38].

Hospital surfaces, medical devices and persistence in the environment play an important role in the dissemination of VREfm in outbreaks [39, 40]. In fact, we also detected outbreak-associated isolates on relevant high-touch surfaces such as an electronic patient chart and computer in the nurses’ station. For Gram-negative multidrug resistant enterobacteria, hospital sink drains have been well-described as potential reservoir, where resistance plasmids are exchanged between different bacterial species causing prolonged outbreaks [41, 42]. So far, less is known about where *vanA* plasmids are exchanged in the hospital environment, i.e. sink drains or surfaces. This question warrants further investigation to improve targeted infection control measures against VREfm in the hospital setting.

Plasmids may play a role in the rapid rise of *vanA* positive isolates in the hospital setting. It seems plausible that the high stability of *vanA* plasmids and the maintenance of more than one *vanA* plasmids leads to a higher capability for adaptation to new and different environments. In fact, our data showed several isolates carrying more than one *vanA* plasmid (*n* = 18). However, larger datasets are necessary to address this question.

## Conclusions

*vanA* plasmid analysis offers an additional dimension to WGS data for outbreak investigations. Furthermore, analysis of *vanA* plasmids in the hospital setting can improve our understanding how VREfm can persist and spread in hospitals.

## Supplementary Information

Additional file 1: Document containing supplementary Table 1 and supplementary Figures 1-3.

## Data Availability

The assemblies of the 72 analyzed VREfm are available at NCBI in the BioProject: BioProject number PRJNA1177408.

## Abbreviations

AMR: Antimicrobial resistance
CC: Clonal complex
CDC: Centers for Disease Control and Prevention
cgMLST: Core genome multilocus sequence typing
CT: Complex type
EARS-Net: European antimicrobial resistance surveillance network
E. faecium: Enterococcus faecium
EUCAST: European Committee on Antimicrobial Susceptibility Testing
HGT: Horizontal gene transfer
IQR: Interquartile range
lrWGS: Long-read whole genome sequencing
MALDI-TOF/MS: Matrix-assisted laser desorption/ionization time-of-flight mass spectrometry
Mash: MinHash: method to compare DNA sequences
MLST: Multilocus sequence typing
NCBI: National Center for Biotechnology Information
ST: Sequence type
UHM: University Hospital Münster
van: A/H/R/S/X/Y/Z vancomycin resistance genes or vanA gene locus
VREfm: Vancomycin-resistant Enterococcus faecium
WGS: Whole genome sequencing
