# Supplementary material for: Characterization of vanA-harboring plasmids supports differentiation of outbreak-related and sporadic vancomycin-resistant Enterococcus faecium isolates in a tertiary care hospital
Source: BMC Microbiol. 2025 May 28;25:337. doi: 10.1186/s12866-025-04058-5 (PMC12117755; doi:10.1186/s12866-025-04058-5)
Supplement: Supplementary file 1 — Additional file 1: Document containing supplementary Table 1 and supplementary Figures 1-3. [file 12866_2025_4058_MOESM1_ESM.docx]

Supplementary Material for:

**Characterization of *vanA*-harbouring plasmids supports differentiation of outbreak-related and sporadic vancomycin-resistant *Enterococcus faecium* isolates**

Authors: A. Sobkowiak^1,2^, N. Scherff^1^, V. van Almsick^1,2^, F. Schuler^3^, T. J. Brix^4^,
A. Mellmann^1*^, V. Schwierzeck^1*^

**Supplementary Figures and Tables**

**Supp. Table 1:** Genetic characteristics of VREfm isolates at the UHM

|  |  | **Overall**, n = 446* | ***vanA***, n = 255 | ***vanB***, n = 189 |
| --- | --- | --- | --- | --- |
| **ST** | 80 | 245 (54.93%) | 217 (85.10%) | 27 (14.29%) |
|  | 117 | 175 (39.24%) | 18 (7.06%) | 156 (82.54%) |
|  | 1299 | 4 (0.90%) | 4 (1.57%) | 0 |
|  | 18 | 4 (0.90%) | 4 (1.57%) | 0 |
|  | other ST | 10 (2.24%) | 7 (2.75%) | 3 (1.59%) |
|  | unknown | 8 (1.79%) | 5 (1.96%) | 3 (1.59%) |
| **CT** | 1470 | 213 (47.76%) | 213 (83.53%) | 0 (0.00%) |
|  | 71 | 114 (25.56%) | 1 (0.39%) | 112 (59.26%) |
|  | 929 | 16 (3.59%) | 9 (3.53%) | 7 (3.70%) |
|  | 1473 | 10 (2.24%) | 0 | 10 (5.29%) |
|  | 1579 | 9 (2.02%) | 0 | 9 (4.76%) |
|  | 6045 | 7 (1.57%) | 0 | 6 (3.17%) |
|  | 2406 | 6 (1.35%) | 0 | 6 (3.17%) |
|  | 8197 | 6 (1.35%) | 0 | 6 (3.17%) |
|  | other CT | 65 (14.57%) | 32 (12.55%) | 33 (17.46%) |

ST: sequence type, CT: cluster type

* n=2 isolates have a combined *vanA*/*vanB* genotype and are stated in the main text

**Supp. Table 2:** Locations of isolation of the environmental isolates

| **Sample ID** | **location of isolation** |
| --- | --- |
| A25769 | medical supply cart |
| A25770 | electronical patient chart |
| A25772 | nursing room: computer, keyboard, phone |
| A25774 | nursing room: desk, keyboard (monitoring system) |
| A25776 | nursing room: chair backs |
| A25778 | laundry storage, positioning cushion |
| A25779 | pharmaceutical refrigerator |
| A25780 | Medical device storage room, monitor (clean), door handles |

**Supp. Table 3:** Epidemiological information on the two largest plasmid clusters

| **Plasmid group** | **Sample ID** | **nosocomial** | **Ward contact** | **Room contact** |
| --- | --- | --- | --- | --- |
| 2 | A29724 | Yes | Yes (2 days) A29350 | No |
| 2 | A33753 | Yes | No | No |
| 2 | A26108 | Yes | No | No |
| 2 | A29350 | Yes | Yes (2 days) A29349 and (2 days) A29724 | No |
| 2 | A29349 | No | Yes (2 days) A29350 | No |
| 2 | A32470 | Yes | No | No |
| 2 | A32468 | Yes | No | No |
| 2 | A32543 | Yes | No | No |
| 2 | A32542 | Yes | No | No |
| 2 | A32592 | Yes | Yes | Yes (3 days) A32790 |
| 2 | A32790 | Yes | Yes | A32592, see above |
| 3 | A29726 | Yes | No | No |
| 3 | A29765 | Yes | Yes | Yes (5 days) A29725 |
| 3 | A28699 | Yes | No | No |
| 3 | A29354 | No | No | No |
| 3 | A29725 | Yes | Yes | A29765, see above |
| 3 | A27687 | Yes | Yes also (1 day) A27904 | Yes (10 days) A27674 |
| 3 | A27674 | Yes | Yes | A27687, see above |
| 3 | A27901 | Yes | Yes (2days) A27904 | No |
| 3 | A27904 | Yes | Yes (1 day) A27687 and (2 days) A27901 | No |
| 3 | A28445 | Yes | No | No |

**Supp. Table 4:** Mash distances between plasmids of different plasmid groups

| **Representative plasmids** | **Plasmid group** | **Mash distance to representative plasmid of group 1** |
| --- | --- | --- |
| p_vanA_A25317 | 1 | - |
| p_vanA_A29724 | 2 | 0.0078 |
| p_vanA_A29726 | 3 | 0.0118 |
| p_vanA_A32287 | 4 | 0.0321 |
| p_vanA_A31008 | 5 | 0.0169 |
| p_vanA_A29264 | 6 | 0.0093 |
| p_vanA_A33124 | 7 | 0.0103 |
| p_vanA_A32287 | 8 | 0.1029 |

**
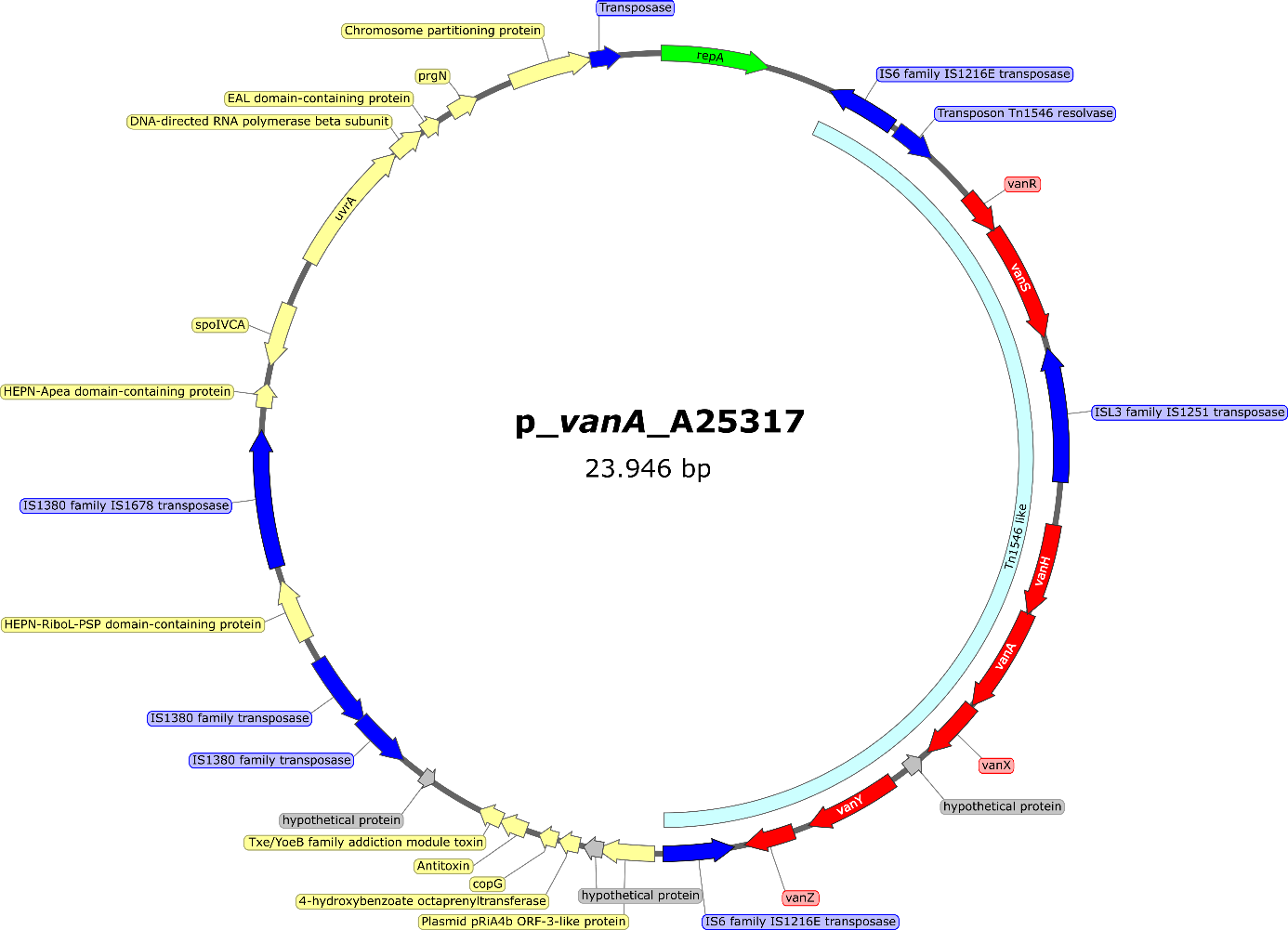
**

**Supp. Figure 1:** Annotation of a representative *vanA* plasmid associated with the described transmission event

Figure shows *vanA* plasmid of the isolate A25317. Gene of the *vanA* gene cluster are illustrated in red, transposases and a resolvase in blue, *repA* in green , other genes in yellow and genes of hypothetical proteins in grey. The Tn1546 like structure is indicated in light blue, but the plasmid is lacking the inverted repeats of known Tn1546.


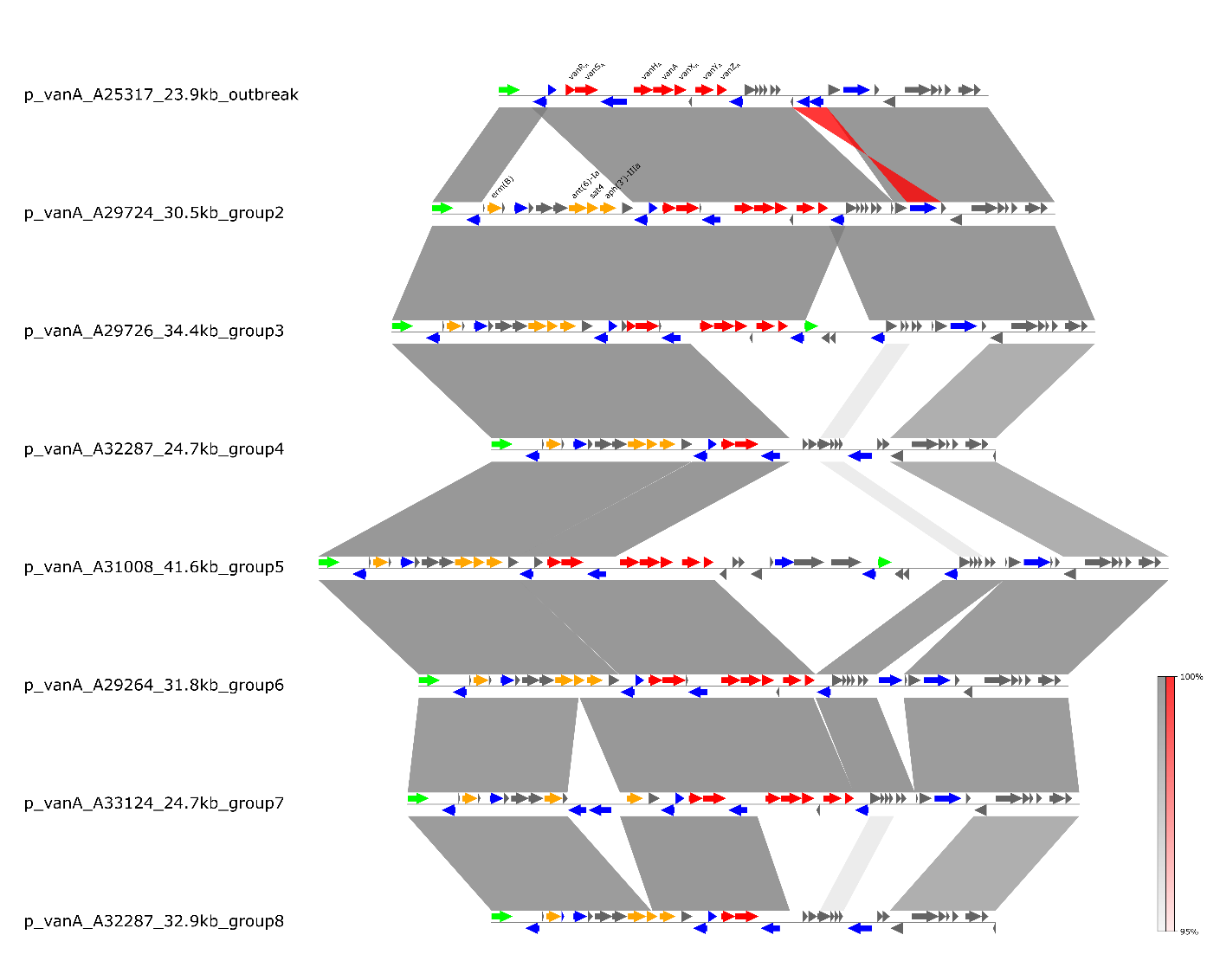
**Supp. Figure 2:** Alignment of representative *vanA* plasmids of plasmid groups 1 to 8.

Figure shows sequence similarities between the outbreak-associated plasmid and representative plasmids of plasmid groups 2 to 8. Intensity of grey boxes indicates degree of similarity and red connections represent an inversion. Genes of the *vanA* gene cluster are illustrated by red arrows. Transposases and a resolvase are shown in blue, *rep* genes in green, other antibiotic resistance genes in orange, and all other genes in grey.

**
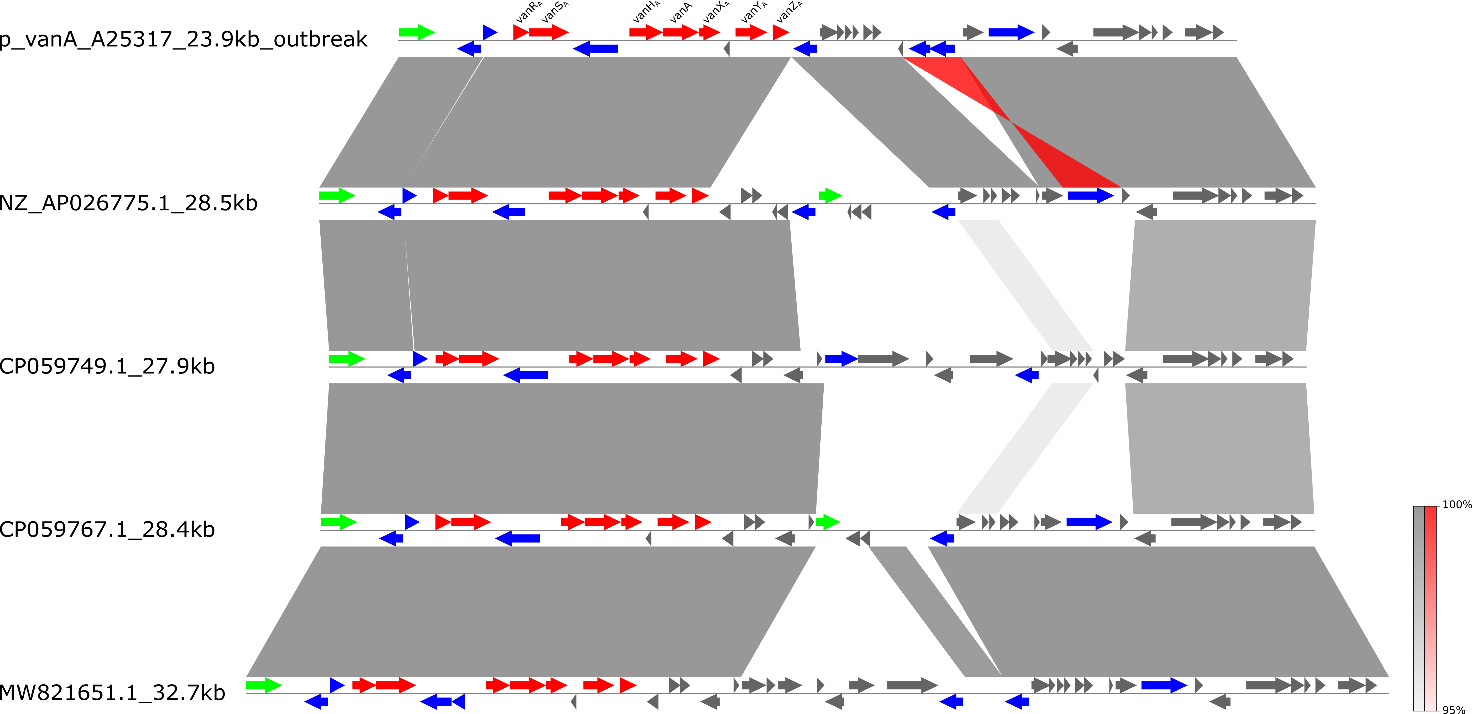
**

**Supp. Figure 3:** Alignment of outbreak associated plasmid with plasmid found in public databases.

Figure shows sequence similarities between the transmission-associated plasmid and first hits in public databases (PLSDB Mash search: NZ_AP026775.1, NCBI Blast search: CP059749.1, CP059767.1, MW821651.1). Intensity of grey boxes indicates degree of similarity and red connections represent an inversion. Genes of the *vanA* gene cluster are illustrated by red arrows, transposases and a resolvase in blue, *rep* genes in green, and all other genes in grey.
